# Validation of the Mind Excessively Wandering Scale and the Relationship of Mind Wandering to Impairment in Adult ADHD

**DOI:** 10.1177/1087054716651927

**Published:** 2016-06-02

**Authors:** Florence D. Mowlem, Caroline Skirrow, Peter Reid, Stefanos Maltezos, Simrit K. Nijjar, Andrew Merwood, Edward Barker, Ruth Cooper, Jonna Kuntsi, Philip Asherson

**Affiliations:** 1 Institute of Psychiatry, Psychology and Neuroscience, King’s College London, UK; 2 Department of Psychology, University of Bath, UK

**Keywords:** ADHD, mind wandering, functional impairment, mental restlessness, task-unrelated thoughts (TUTs)

## Abstract

**Objective:** This study investigates excessive mind wandering (MW) in adult ADHD using a new scale: the Mind Excessively Wandering Scale (MEWS). **Method:** Data from two studies of adult ADHD was used in assessing the psychometric properties of the MEWS. Case-control differences in MW, the association with ADHD symptoms, and the contribution to functional impairment were investigated. **Results:** The MEWS functioned well as a brief measure of excessive MW in adult ADHD, showing good internal consistency (α > .9), and high sensitivity (.9) and specificity (.9) for the ADHD diagnosis, comparable with that of existing ADHD symptom rating scales. Elevated levels of MW were found in adults with ADHD, which contributed to impairment independently of core ADHD symptom dimensions. **Conclusion:** Findings suggest excessive MW is a common co-occurring feature of adult ADHD that has specific implications for the functional impairments experienced. The MEWS has potential utility as a screening tool in clinical practice to assist diagnostic assessment.

## Introduction

The diagnosis of ADHD is based mainly on descriptions of behaviors that reflect inattention, hyperactivity, and impulsivity. Yet older children, adolescents, and adults frequently report phenomenological descriptions of internal subjective experiences that may underlie the behavioral changes seen in ADHD. Characteristic descriptions of the mental state in ADHD include reports of ceaseless mental activity, thoughts that are constantly on the go, or a mind constantly full of thoughts. Thoughts are experienced as uncontrolled, with multiple occurring at the same time. Another common description is of short-lived thoughts that flit from one thing to another, jumping between different ideas (Asherson, 2005; Downey, Stelson, Pomerleau, & Giordani, 1997; Weyandt et al., 2003). Here, we propose that such excessive mind wandering (MW) may reflect a core difficulty in ADHD that underlies some of the experienced impairments.

MW is conceptualized as periods in time when attention and the contents of thoughts shift away from external sources and/or ongoing tasks to unrelated internal thoughts or feelings (Smallwood & Schooler, 2015). It is a universal human experience; individuals in the general population are estimated to spend between 24% and 50% of their waking hours engaging in self-generated thoughts unrelated to their external environment (Kane et al., 2007; Killingsworth & Gilbert, 2010; Smallwood & Schooler, 2015; Song & Wang, 2012). Two main types of MW have been identified; first, self-generated internal thoughts that occur intentionally/deliberately, such as planning the menu for a party while driving to work. Second, unintentional/spontaneous MW when the mind drifts off, for example, during a lecture or conversation. Despite its ubiquitous nature, individuals differ in the frequency and intentionality of their MW.

Excessive spontaneous MW has been associated with functional impairment and implicated in psychopathologies such as ADHD (Franklin et al., 2017). Mental restlessness, a descriptive term encompassing excessive MW, has been reported as more common in ADHD than non-ADHD individuals (Downey et al., 1997; Weyandt et al., 2003). Previous work suggests that ADHD is associated with spontaneous MW, rather than deliberate MW, and detrimental episodes of MW (Franklin et al., 2017; Seli, Smallwood, Cheyne, & Smilek, 2015; Shaw & Giambra, 1993). Detrimental MW has been defined as instances when task-unrelated thoughts (TUTs) interfere with task performance. In contrast, strategic MW occurs at times when TUTs are less likely to interfere with performance (whether intentional or not) or when the benefits outweigh the costs, and can be an economic use of neuronal resources (Franklin et al., 2017; Smallwood & Schooler, 2015).

Using an experience sampling technique to measure on- and off-task thoughts during an attention task, Shaw and Giambra (1993) found the frequency of spontaneous (but not deliberate) TUTs was increased in college students with a childhood history of ADHD compared with controls. Furthermore, a sub-clinical group with high levels of ADHD symptoms demonstrated more TUTs compared with those with low ADHD scores. This finding was subsequently replicated using a rating scale measure of deliberate and spontaneous MW in both clinical and non-clinical ADHD samples (Seli et al., 2015). In addition, regression analyses revealed spontaneous MW to be independently related to ADHD symptomatology, whereas deliberate MW was unrelated, further suggesting that spontaneous MW is a feature of ADHD.

ADHD symptomatology has also been shown to positively correlate with both the frequency of MW and the lack of awareness of engaging in MW (Franklin et al., 2017). A sub-clinical group with high ADHD symptom scores had disruptive MW episodes even when they were detrimental and interfered with function in daily life. In this study, lacking awareness of MW was shown to mediate between ADHD symptoms and impairment, suggesting that increasing awareness of MW in ADHD might lead to functional improvements.

**Figure 1.**
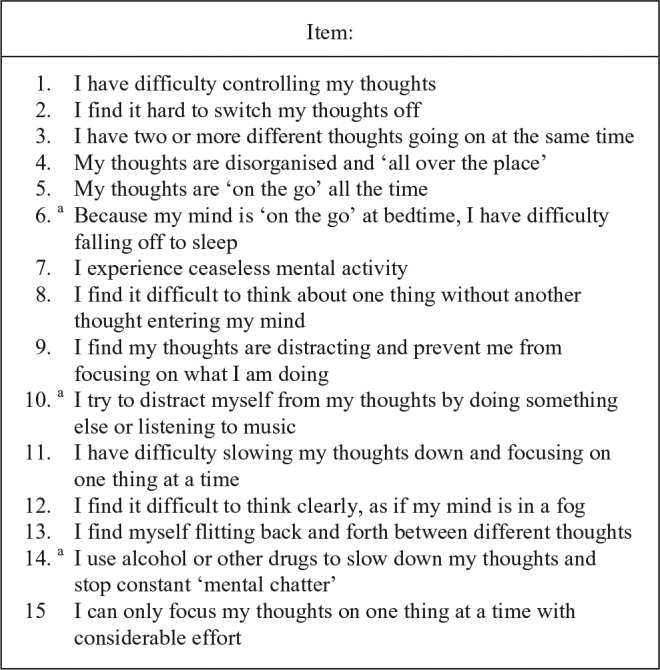
Items from the Mind Excessively Wandering Scale (MEWS). *Note.* Items are scored on a 4-point Likert-type scale (0 = *not at all* or *rarely*, 1 = *some of the time*, 2 = *most of the time*, 3 = *nearly all of the time* or *constantly*). The MEWS scale is copyrighted and available without charge from the corresponding author, and we welcome use of this scale in research. ^a^Items we recommend excluding from the scale in future research, based on analysis conducted here.

Collectively, these findings suggest that adults with ADHD are highly susceptible to excessive spontaneous MW and may have a core difficulty controlling spontaneous thoughts unrelated to the current context. Excessive MW could therefore underlie many of the symptoms and impairments that characterize the disorder. To explore the role that MW may play in the pathogenesis of ADHD, as well as its potential role in diagnosis, our research group developed the Mind Excessively Wandering Scale (MEWS; see Figure 1). The MEWS is a 15-item self-report measure designed to reflect MW in ADHD, derived from patient reports of subjective experiences of their thought processes. The scale captures the main characteristics of the mental state described by adults with ADHD: thoughts on the go all the time, thoughts that jump or flit from one topic to another, and multiple lines of thoughts at the same time (Asherson, 2005). The MEWS therefore reflects the form as opposed to the content of the experienced thought processes in ADHD. Uniquely, the MEWS assesses a mental phenomenon as opposed to the behavioral symptoms conventionally assessed with ADHD rating scales.

The aim of the present study was to validate the MEWS as an instrument to assess MW in adult ADHD using two study samples. In Study 1, we conducted a preliminary evaluation of the psychometric properties of the MEWS in a small sample of adult males with ADHD selected for the absence of comorbid psychiatric conditions. In Study 2, we cross-validated the MEWS in a larger independent sample including males and females, less highly selected against comorbidity. We further investigated the relationship of MEWS scores to other measures of ADHD symptomatology, and investigated the relationship between MW and functional impairment.

## Method

### Study 1 Sample

Participants were a small subset of adults from the MIRIAD (Mood Instability Research in ADHD) project, a longitudinal case-control study of emotional lability (EL) and neuropsychological functioning in adult men with ADHD with no co-occurring comorbidities (Skirrow & Asherson, 2013). Forty-one adults with ADHD and 47 controls aged between 18 and 65 years (ADHD: *M* = 28.54 years, *SD* = 9.52 years; control: *M* = 29.00 years, *SD* = 10.46 years) participated in the MIRIAD project. There were no significant differences between groups for age or IQ (see Table 1). ADHD participants were recruited from the waiting list of the National Adult ADHD Clinic at the South London and Maudsley Hospital (SLaM) and were medication free at the time of the research assessment. Further detail on the recruitment process is provided elsewhere (Skirrow & Asherson, 2013).

**Table 1.** Case-Control Differences for Age, Sex, IQ, MEWS, INN, HI, EL, and IMP.

	Study 1							Study 2						
	ADHD			Control			*p*	ADHD			Control			*p*
	*N*	*M*	*SD*	*N*	*M*	*SD*		*N*	*M*	*SD*	*N*	*M*	*SD*	
Age	41	28.54	9.52	47	29.00	10.46	.83	81	33.52	10.26	30	29.51	8.80	.06
Male	41			47			—	44			16			.93
Female	0			0				37			14			
IQ	41	108.95	15.08	47	113.15	13.36	.17	80	109.38	13.68	23	111.59	11.62	.44
Time 1														
MEWS	25	25.00	10.11	24	4.79	6.98	<.0001	79	27.72	9.31	29	7.21	6.26	<.0001
INN	41	19.34	5.03	47	3.87	3.44	<.0001	81	27.16	6.13	30	6.23	3.99	<.0001
HI	40	16.20	6.59	47	3.28	3.30	<.0001	81	20.09	5.80	30	5.33	4.08	<.0001
EL	40	45.93	11.76	46	25.24	9.17	<.0001	80	24.31	12.09	30	4.17	4.86	<.0001
IMP	41	1.23	0.41	47	0.30	0.28	<.0001	80	1.22	0.49	30	0.20	0.20	<.0001

*Note.* See Online Supplementary Table 1 for Time 2 and Time 3. MEWS = Mind Excessively Wandering Scale; INN = inattention; HI = hyperactivity/impulsivity; EL = emotional lability; IMP = impairment.

As the MEWS was developed after the MIRIAD project began, only a subset of the ADHD cases and controls provided MEWS data. At study entry (baseline), 25 cases and 24 controls completed the MEWS. Follow-up assessments completed approximately 9 months after baseline provided data on 18 cases and 18 controls at both time points. In addition, six cases and 18 controls provided MEWS data at follow-up assessment alone. Of the 18 ADHD cases with data at both time points, 16 were treated with methylphenidate and one with atomoxetine at follow-up, initiated by local services and not following a specific protocol. Ethical approval for this study was obtained from the Joint Research Ethics Committee of the Institute of Psychiatry and SLaM.

### Study 2 Sample

Participants were from the OCEAN (Oils and Cognitive Effects in Adult ADHD Neurodevelopment) project, a study investigating the relationship of omega-3 dietary supplementation (not analyzed in this study) to cognitive and electrophysiological measures in adults with ADHD. Participants were aged between 18 and 65 years. The sample consisted of 81 adults with ADHD (37 female, 44 male; *M* age = 33.52 years, *SD* = 10.26 years) and 30 healthy controls (14 female, 16 male; *M* age = 29.51 years, *SD* = 8.8 years). Groups did not significantly differ on age, sex, or IQ (see Table 1). ADHD participants were recruited through SLaM Adult ADHD Service, advertisements on ADHD support websites, and previous study databases. See online supplementary material for further information on recruitment.

At baseline (Time 1), 79 cases and 29 controls provided MEWS data. Two separate follow-up assessments of the ADHD cases took place, 3 months (Time 2) and 6 months (Time 3) after baseline. At Time 2 and Time 3, 79 and 55 ADHD cases provided MEWS data, respectively. Ethical approval for the study was granted by the National Research Ethics Service (NRES) Committee London.

### Measures

#### ADHD symptoms

ADHD symptoms were assessed using the self-rated Barkley Adult ADHD Rating Scale (BRS; Barkley, 1998) in Study 1, and the Conners’ Adult ADHD Rating Scales (CAARS; Conners, Erhardt, & Sparrow, 1999) in Study 2. Both scales cover the same list of 18 DSM-IV/DSM-5 items for inattention and hyperactivity (*Diagnostic and Statistical Manual of Mental Disorders* (4th ed. and 5th ed.; American Psychiatric Association [APA], 1994 and 2013).

#### Emotional dysregulation

Emotional Lability (EL) was measured using the Affective Lability Scale–Short Form (ALS-SF; Oliver & Simons, 2004), which measures rapid changes in emotional states.

#### Impairment

Functional impairment across major life domains (family, work, school, life-skills, self-concept, social, and risk) was measured using the Weiss Functional Impairment Rating Scale–Self-Report (WFIRS-S; Sadek, 2014).

#### Mind wandering

MW was measured using the newly created MEWS (see Figure 1). This publication is the first report of this scale. The MEWS is a 15-item self-report measure reflecting MW in ADHD. Items were based on patient descriptions of MW in ADHD as previously described by Asherson (2005). P.A., C.S., and P.R. drew up the list of questions based heavily on their combined experience of patient’s reports of MW, and questions were refined during several consensus meetings. The final item checklist was agreed by all three authors and implemented initially in the MIRIAD study before further testing in the OCEAN study (reported here). The MEWS scale is copyrighted and available without charge from the corresponding author.

### Statistical Analyses

Mean values for each rating scale and subscale were used as summary measures. The raw data and square-root transformations were used in analysis, and parametric and non-parametric tests were used as appropriate.

Principal components analysis (PCA) with varimax rotation was conducted to examine the factor structure of the MEWS. Cronbach’s alpha was used as a measure of reliability to assess internal consistency and Pearson’s correlation coefficient was used to analyze test–retest reliability of the scale. Construct validity was assessed with independent *t* tests and Mann–Whitney U tests to investigate case-control differences. Receiver operating characteristic (ROC) analysis was used to examine diagnostic accuracy and the optimal cut-off point of the measure.

Convergent validity of the MEWS in relation to ADHD symptom scales was assessed using polyserial correlations to provide unbiased estimates of cross-variable correlations in case-control samples (Olsson, 1979). For these analyses, we fixed the *z* value threshold for affection status corresponding to 3.4% prevalence of ADHD in adults (Fayyad et al., 2007). In Study 1, polyserial correlations were also conducted on change scores (Time 1-Time 2). For change scores in Study 2, we used partial correlations to control for potential influences of the study intervention (placebo or essential fatty acid). Hierarchical multiple regression was used to investigate whether MEWS scores were independent predictors of impairment; inattention and hyperactivity/impulsivity were entered in the first step and MW in the second.

## Results

### Study 1

#### Psychometric evaluation

The scree plot and eigenvalues indicated a unidimensional structure to the MEWS with one factor accounting for 69.16% of the variance (eigenvalue = 10.37; see online supplementary material). Factor loadings were greater than .7, with the exception of Item 14 (.51). Table 2 shows Cronbach’s alpha coefficients for the full 15-item MEWS in comparison with the other rating scales. At baseline, internal consistency was high for all scales for both cases and controls (α > .78). Examination of item total correlations showed each item to correlate well with the full 15-item scale (correlations > .75, with the exception of Items 6 [.66], 10 [.70], and 14 [.47]), suggesting items are measuring the same underlying construct. Inter-item correlations ranged from .27 to .88, with an average inter-item correlation of .66, reflecting the internal consistency of the scale items.

**Table 2.** Reliability Coefficients (α) for the MEWS as Compared With the INN, HI, EL, and IMP Rating Scales.

Study 1	Time 1			Time 2		
	Whole sample	Cases	Controls	Whole sample	Cases	Controls
MEWS	.97	.93	.95	.96	.94	.93
INN	.96	.83	.80	.96	.92	.82
HI	.95	.88	.78	.92	.88	.76
EL	.96	.91	.95	.96	.91	.96
IMP	.98	.96	.96	.97	.88	.98
Study 2	Time 1			Time 2	Time 3	
	Whole sample	Cases	Controls	Cases	Cases	
MEWS	.96	.91	.90	.94	.95	
INN	.96	.85	.82	.91	.98	
HI	.93	.83	.75	.87	.99	
EL	.95	.92	.87	.91	.93	
IMP	.98	.98	.86	.98	.97	

*Note.* MEWS = Mind Excessively Wandering Scale; INN = inattention; HI = hyperactivity/impulsivity; EL = emotional lability; IMP = impairment.

There was a mean interval of 9.7 months (*SD* = 3.3 months) for cases and 9.5 months (*SD* = 4.0 months) for controls between baseline and follow-up. Test–retest reliability was significant for the whole sample (*r* = .84, 95% confidence interval [CI] = [.74, .92], *p* < .001), and for both cases (*r* = .63, 95% CI = [.06, .88], *p* = .005) and controls (*r* = .82, 95% CI = [.40, .93], *p* < .001).

#### Construct validity

Case-control comparisons at baseline revealed significantly elevated ratings of MW in individuals with ADHD, *t*(47) = −7.83, *p* < .0001, comparable with that found for the other rating scales of ADHD symptom domains: inattention, *t*(73.07) = −14.58, *p* < .0001; hyperactivity/impulsivity, *t*(85) = −11.40, *p* < .0001; emotional lability, *U* = 168.5, *z* = −6.53, *p* < .0001 (Table 1). Participants with ADHD also demonstrated significantly greater overall impairment on the WFIRS-S, *t*(86) = −13.08, *p* < .0001, as well as for each domain of impairment, *t* range = −5.78-11.40, *p* < .0001, for impairment in family life, work, school, life-skills, self-concept, social problems, and risk taking. Similar results were found at follow-up (see online supplementary material).

ROC analysis was used to examine the capacity of the scale to discriminate between cases and controls. Area under the curve (AUC) was .92 (95% CI = [.85, 1.00], *p* < .0001) which, being close to 1, indicates excellent discriminant capacity of the MEWS. This was comparable with the AUC value of existing rating scales of ADHD symptom domains (inattention: AUC = .99, 95% CI = [.97, 1.00]; hyperactivity/impulsivity: AUC = .95, 95% CI = [.91, .99]; emotional lability: AUC = .91, 95% CI = [.84, .97]). A score of 15 or above provides the optimal balance of sensitivity (.88) and specificity (.88), suggesting a cut-off for disorder threshold (see online supplementary material).

#### Convergent validity

Polyserial correlations in the combined case-control data set showed strong positive correlations between MW and the other rating scales of ADHD and impairment: inattention (*r* = .81, 95% CI = [.72, .87]), hyperactivity/impulsivity (*r* = .77, 95% CI = [.66, .84]), emotional lability (*r* = .81, 95% CI = [.72, .88]), and impairment (*r* = .82, 95% CI = [.71, .89]), as well as ADHD affection status (*r* = .70, 95% CI = [.57, .79]). The strongest correlation was between MW and impairment (Table 3). In addition, moderate to large positive correlations were seen between MW and ADHD symptom dimensions and impairment in both cases and controls analyzed separately (see online supplementary material), indicating severity of symptoms and impairment in both cases and controls.

**Table 3.** Polyserial Correlations (95% Confidence Intervals), Corrected for Selection (Affection Threshold = 3.4%), Between the MEWS, INN, HI, EL, and IMP Rating Scales, and AFF.

Time 1	MEWS	INN	HI	EL	IMP
Study 1					
MEWS	—	—	—	—	—
INN	.81 [.72, .87]	—	—	—	—
HI	.77 [.66, .84]	.85 [.79, .89]	—	—	—
EL	.81 [.72, .88]	.74 [.65, .81]	.75 [.64, .81]	—	—
IMP	.82 [.71, .89]	.83 [.76, .88]	.82 [.75, .87]	.77 [.68, .83]	—
AFF	.70 [.57, .79]	.83 [.75, .89]	.71 [.60, .79]	.65 [.51, .75]	.75 [.64, .83]
Study 2					
MEWS	—	—	—	—	—
INN	.77 [.69, .83]	—	—	—	—
HI	.69 [.58, .76]	.76 [.68, .82]	—	—	—
EL	.74 [.66, .81]	.62 [.50, .71]	.53 [.40, .65]	—	—
IMP	.81 [.74, .086]	.74 [.65, .80]	.65 [.54, .73]	.78 [.70, .83]	—
AFF	.67 [.55, .77]	.83 [.76, .88]	.74 [.64, .81]	.59 [.46, .70]	.68 [.57, .78]

*Note.* MEWS = Mind Excessively Wandering Scale; INN = inattention; HI = hyperactivity/impulsivity; EL = emotional lability; IMP = impairment; AFF = affection status.

For the sub-sample with both baseline and follow-up data, the correlation of baseline to follow-up change scores for MW with change scores for the rating scale measures of ADHD symptoms and impairments revealed temporal covariance between the measures: positive correlations were found between change in MW and change in inattention (*r* = .72, 95% CI = [.54, .83]), hyperactivity/impulsivity (*r* = .55, 95% CI = [.33, .70]), emotional lability (*r* = .70, 95% CI = [.44, .84]), and impairment (*r* = .51, 95% CI = [.23, .72]; see Table 4). In the 16 cases treated with methylphenidate at follow-up, there was a significant reduction in MEWS scores, *t*(15) = 2.28, *p* = .04, between the baseline medication-free period (*M* = 23.38, *SD* = 10.93) and follow-up (*M* = 17.25, *SD* = 10.58).

**Table 4.** Correlations (95% Confidence Intervals) Between Change Scores for the MEWS, INN, HI, EL, and IMP Rating Scales.

Change scores	MEWS	INN	HI	EL
Study 1				
MEWS	—	—	—	—
INN	.72 [.54, .83]	—	—	—
HI	.55 [.33, .70]	.73 [.59, .82]	—	—
EL	.70 [.44, .84]	.55 [.35, .70]	.58 [.39, .72]	—
IMP	.51 [.23, .72]	.62 [.45, .74]	.62 [.45, .74]	.55 [.35, .70]
Study 2				
MEWS	—	—	—	—
INN	.53 [.25, .71]****	—	—	—
HI	.31 [.01, .52]*	.74 [.53, .86]****	—	—
EL	.43 [.19, .62]***	.48 [.16, .71]****	.31 [−.03, .59]*	—
IMP	.62 [.37, .78]****	.50 [.31, .66]****	.41 [.18, .59]**	.44 [.18, .65]***

*Note*. Polyserial correlations corrected for selection (affection threshold = 3.4%) were used in Study 1, and partial correlations to correct for study intervention in Study 2 (with significance levels). MEWS = Mind Excessively Wandering Scale; INN = inattention; HI = hyperactivity/impulsivity; EL = emotional lability; IMP = impairment.

* *p* < .03. **p < .01. ***p = .001. ****p < .0001.

#### Impairment

Data from 49 participants were used in regression analyses with the WFIRS-S total impairment score. Inattention and hyperactivity/impulsivity accounted for 82.4% of the variability in functional impairment (*R*^2^ = .824). The addition of MW as a predictor led to a significant increase in predictive power of the model (*R*^2^Δ = .024), with the variability accounted for by the model increasing to 84.9%, *F*Δ(1, 45) = 7.17, *p* = .01. This indicates that MW is having a small but significant effect beyond that accounted for by inattention and hyperactivity/impulsivity. Inattention carried the most importance in the model (β = .45), followed by MW (β = .31) and hyperactivity/impulsivity (β = .21). Only inattention and MW significantly contributed to the model (*p* = .001 and .01, respectively).

Within the ADHD group, MW had an independent effect on impairment in the domains of self-concept, *R*^2^Δ = .145, *F*Δ(1, 21) = 6.57, *p* < .02, and social problems, *R*^2^Δ = .137, *F*Δ(1, 21) = 4.50, *p* < .05. For the social problems domain, MW carried more importance in the model (β = .44) than inattention (β = −.42), but not hyperactivity/impulsivity (β = .50). Only MW and hyperactivity/impulsivity significantly contributed to the model (*p* = .05 and .03, respectively). Interpretation of work and life-skills dimensions was not possible due to heteroskedasticity in the data.

### Study 2

#### Psychometric evaluation

The scree plot and eigenvalues suggested a one-factor solution with an eigenvalue of 9.44, accounting for 62.92% of the variance (see online supplementary material). All items loaded highly onto this factor (>.76, with the exception of Items 6 [.60], 10 [.68], and 14 [.40]). Internal consistency was high for all scales for both cases and controls (α > .78; see Table 2). Examination of item total correlations showed each item to correlate well with the full 15-item scale (correlations > .72, with the exception of Items 6 [.56], 10 [.63], and 14 [.37]). Inter-item correlations ranged from .24 to .82, with an average inter-item correlation of .59, reflecting the internal consistency of the scale items.

The mean interval between Time 1 and Time 3 was 6.4 months (*SD* = 0.58 months), and MEWS scores showed satisfactory retest reliability across this time period (*r* = .63, 95% CI = [.42, .80], *p* < .0001).

#### Construct validity

Case-control comparisons revealed significantly elevated ratings of MW in individuals with ADHD (*U* = 87.00, *z* = −7.34, *p* < .0001). This difference was comparable with that found for the other rating scales of ADHD symptom domains (inattention: *U* = 20.00, *z* = −7.94, *p* < .0001; hyperactivity/impulsivity: *U* = 64.50, *z* = −7.65, *p* < .0001; emotional lability: *U* = 140.50, *z* = −7.12, *p* < .0001; see Table 1). ADHD cases also demonstrated significantly greater overall impairment on the WFIRS-S (*U* = 53.50, *z* = −7.70, *p* < .0001), as well as for each domain of impairment (*z* range = −4.87 to −7.57, *p* < .0001 for impairment in family life, work, school, self-concept, social problems, life-skills, and risk taking; see online supplementary material).

ROC curve analysis indicated that the MEWS successfully discriminated between cases and controls (AUC = .96, 95% CI = [.93, .99], *p* < .0001). This was comparable with the AUC value of existing rating scales of ADHD symptom domains (inattention: AUC = .99, 95% CI = [.98, 1.00]; hyperactivity/impulsivity: AUC = .97, 95% CI = [.95, 1.00]; emotional lability: AUC = .94, 95% CI= [.90, .98]). A score of 15 on the MEWS provides the optimal balance of sensitivity (.90) and specificity (.90; see online supplementary material).

#### Convergent validity

Using polyserial correlations in the combined case-control data set, we found a positive correlation between MW and the other rating scales of ADHD and impairment: inattention (*r* = .77, 95% CI = [.69, .83]), hyperactivity/impulsivity (*r* = .69, 95% CI = [.58, .76]), emotional lability (*r* = .74, 95% CI = [.66, .81]), impairment (*r* = .81, 95% CI = [.74, .86]), and ADHD affection status (*r* = .67, 95% CI = [.55, .77]). The strongest correlation was between MW and impairment (see Table 3). Moderate to large positive correlations were also seen between MW and ADHD symptom dimensions and impairment in both cases and controls analyzed separately (see online supplementary material).

Investigation of change scores also revealed a temporal relationship. Analyses indicated significant covariation of change in MW with change in inattention (*r* = .53, 95% CI = [.25, .71], *p* < .0001), hyperactivity/impulsivity (*r* = .31, 95% CI = [.01, .52], *p* = .02), emotional lability (*r* = .43, 95% CI = [.19, .62], *p* = .001), and impairment (*r* = .62, 95% CI = [.37, .78], *p* < .0001). MW and impairment showed the strongest relationship (see Table 4).

#### Impairment

Data from 108 participants were used in regression analysis with the WFIRS-S total impairment score. Inattention and hyperactivity/impulsivity accounted for 71.3% of the variability in functional impairment (*R*^2^ = .713). The addition of MW as a predictor led to a significant increase in predictive power of the model (*R*^2^Δ = .076), with the variability accounted for by the model increasing to 78.9%, *F*Δ(1, 104) = 37.17, *p* < .0001. MW carried the most importance in the model (β = .49), followed by inattention (β = .29) and hyperactivity/impulsivity (β = .17). Only MW (*p* < .0001) and inattention (*p* = .002) significantly contributed to the model.

Within the ADHD group, MW had an independent effect on impairment in life-skills, *R*^2^Δ = .18, *F*Δ(1, 75) = 24.79, *p* < .0001; self-concept, *R*^2^Δ = .10, *F*Δ(1, 75) = 9.72, *p* = .003; social problems, *R*^2^Δ = .10, *F*Δ(1, 75) = 9.92, *p* = .002; and risk taking, *R*^2^Δ = .09, *F*Δ(1, 75) = 9.37, *p* = .003. MW carried the most importance in the model for life-skills (β = .52), self-concept (β = .39), and social problems (β = .39), and was the only significant contributor to the model for the self-concept (*p* = .003) and social problems (*p* = .002) domains. Interpretation of the family and work domains was not possible due to heteroskedasticity in the data.

## Discussion

We report the psychometric properties and initial validation findings for a new self-report scale of excessive MW in adults with ADHD. Using two independent samples, we found that MEWS scores functioned extremely well as a measure of the mental phenomenon of MW in ADHD, with good reliability and high sensitivity and specificity for ADHD case-control differences. We found that elevated levels of MW (as indexed by the MEWS) in participants with ADHD were related to self-report measures of functional impairment. Furthermore, the contribution of MW to impairment was independent of the core ADHD symptoms of inattention and hyperactivity/impulsivity. These findings suggest that excessive MW is a characteristic feature of adult ADHD that has specific effects on impairment.

Principal components analysis indicated a unidimensional structure to the scale and other psychometric properties of the MEWS were comparable with existing rating scales of ADHD core symptoms, including good internal consistency and test–retest reliability. The MEWS was able to differentiate between those with and without ADHD with high sensitivity and specificity of the scale, using a threshold score of 15. This is remarkable given that patients were selected for high ADHD symptoms and not specifically for subjective reports of internal thought processes as measured by the MEWS.

In both studies, item total correlations with the full 15-item scale and factor loadings were high apart from Items 6 (Because my mind is “on the go” at bedtime, I have difficulty falling off to sleep), 10 (I try to distract myself from my thoughts by doing something else or listening to music), and 14 (I use alcohol or other drugs to slow down my thoughts and stop constant “mental chatter”). This is likely explained by the nature of these items, which refer to how individuals cope with MW or how it directly affects their functioning, as opposed to a description of the mental phenomenon. To investigate whether the scale could be shortened by dropping Items 6, 10, and 14 without reducing its sensitivity and specificity, we repeated the ROC analysis, finding the shorter 12-item scale had a sensitivity of .89 and specificity of .90 (see online supplementary material). Further analyses in larger data sets could be used to further refine the scale, but based on these data we recommend that future studies use the reduced 12-item scale.

Our findings are in line with previous studies which report elevated levels of MW in ADHD compared with controls, whether measured using clinical rating scales (Franklin et al., 2017; Seli et al., 2015; Weyandt et al., 2003) or experience sampling of TUTs during a sustained attention task (Shaw & Giambra, 1993). Furthermore, the strength of case-control differences for MEWS scores was comparable with that seen for rating scale measures of core ADHD symptoms, for which clinical cases of ADHD are selected on. MEWS scores were also found to be highly correlated with ADHD symptoms and impairment in the total sample, as well as in cases and controls analyzed separately, replicating previous studies of the association between spontaneous MW and ADHD (Franklin et al., 2017; Seli et al., 2015). These results indicate that the MEWS is a marker of symptom severity in both cases and controls, in line with previous data indicating that ADHD symptoms lie along a continuum in the general population (Chen et al., 2008; Salum et al., 2014).

Change scores for MW also covaried with ADHD symptoms and impairments over time, indicating a close temporal relationship consistent with a potential causal role of MW in ADHD. The finding of significant pre–post treatment effects of methylphenidate in a subset of Study 1 participants raises the possibility that treatment effects on ADHD might be mediated by reductions in MW. However, we were unable to test specifically for treatment effects of methylphenidate because we did not randomize to treatment or include a placebo control arm.

The link between MW and impairment was particularly strong, indicating the clinical importance of MW in adults with ADHD. Of specific interest was the finding that MW showed a main effect on impairment beyond the influence of inattention and hyperactivity/impulsivity and was overall the strongest predictor of impairment in Study 2. Investigating specific domains of impairment, MW was found to be an independent predictor of self-concept and social problems in both studies, and additionally life-skills and risk taking in Study 2. The reasons the MEWS is a particularly good predictor of impairment in ADHD are not well understood, but could be explained by both clinical and theoretical considerations. One possible explanation is that the scale items are rooted in qualitative accounts from adult ADHD patients of experiences of their mental state. When asked to describe the subjective experience of the flow of their thoughts, adults with ADHD repeatedly give descriptions of ceaseless, short-lived, and unfocused thoughts that flit from one topic to another (Asherson, 2005). Such a distractible and poorly regulated mental state could be impairing for several reasons.

First, excessive MW may have a specific effect on functional outcomes due to the failure to deal with distraction and deficient mental processing of “task”relevant events. In social situations, an individual with excessive MW may miss verbal and non-verbal information and effectively *not listen* or *lack awareness* of social cues. MW may make it difficult to follow a single line of thought and interrupting others during conversations could be a strategy to avoid losing their train of thought. Behaviors such as these are likely to have negative effects on an individual’s social interactions.

Second, lack of attention paid to events due to one’s mind constantly being “on the go” in a non-focused way can also create difficulties with thinking through and planning activities, linked to forgetfulness and disorganization and leading to impairments in basic life-skills. Impaired self-concept may then arise as a bi-product of the effect of excessive MW on other domains of functioning, but could also be due to distress from the constant effort to focus or the experience of having a mind constantly full of unfocused distractible thoughts. Many patients report a sense of calm and relief when the flow of their thoughts becomes more focused and regulated following stimulants or other treatments for ADHD.

Third, the connection between MW and risk-taking behavior is less obvious, but could be due to the impact of highly salient activities, which engage the attention of individuals, leading to a reduction of spontaneous MW and a sense of relief. For the same reasons, some patients with even severe levels of ADHD may excel at activities such as exciting/stimulating sports. Although there is as yet no direct evidence for this hypothesis, studies investigating default mode deactivation (Liddle et al., 2011) and reaction time variability (RTV; Andreou et al., 2007) during tasks requiring sustained attention have shown reduced or absent case-control differences when conducted under highly salient conditions. Reductions in default mode activity under rewarding conditions have been hypothesized to reflect reductions in excessive MW (Liddle et al., 2011). Thus, risky behavior may reflect individuals seeking out activities with salient content, which decreases MW and helps individuals with ADHD to focus their attention.

These accounts of MW leading to impairment in ADHD remain speculative because of the lack of research on MW in ADHD. However, an increase in understanding of MW states in control participants provides a strong theoretical basis for the hypothesis that excessive MW may underlie many of the behavioral symptoms and impairments seen in ADHD. In healthy control samples, MW is associated with performance deficits that overlap with impairments seen in ADHD, including educational performance, driving accidents, and performance on cognitive tasks including errors of commission and RTV during sustained attention and inhibition tasks (Smallwood & Schooler, 2015). Understanding of the neural processes involved in the regulation of internal thought, involving default mode network (DMN) and executive control networks, has advanced in recent years, and overlaps with neural mechanisms implicated in ADHD. TUTs are strongly associated with deficient task-induced deactivation of the DMN (correlation about .9; McKiernan, D’Angelo, Kaufman, & Binder, 2006), and deficient DMN deactivation during task conditions is strongly associated with ADHD (Christakou et al., 2013). Spontaneous MW that is detrimental to performance has, therefore, been proposed as a mechanism that explains many of the symptoms and functional impairments of ADHD (Seli et al., 2015; Weyandt et al., 2003), reflecting aberrant inter-relationships between default and task positive networks (Fox, Spreng, Ellamil, Andrews-Hanna, & Christoff, 2015; Sripada, Kessler, & Angstadt, 2014).

Interestingly, in one study, meta-awareness of MW (being aware that your mind has wandered) was found to mediate the relationship between ADHD symptoms and detrimental forms of MW, suggesting that psychological treatments aimed at enhancing meta-awareness of MW, such as mindfulness-based interventions (MBIs), might ameliorate the negative consequences of MW in ADHD (Franklin et al., 2017). Recent studies support the beneficial effects of MBIs on ADHD, with the largest study to date showing an effect of *d* = .85 on ADHD symptoms compared with a treatment as usual group (Hepark et al., 2019). Future large-scale controlled experimental designs are therefore indicated to investigate the potential role of MW as a treatment target for the control of ADHD symptoms and impairments using both pharmacological and non-pharmacological interventions.

Current screening tools for adult ADHD consist of rating scales for inattention and hyperactivity/impulsivity. Our findings suggest potential utility of the MEWS as an additional screening tool for adult ADHD in clinical practice, particularly as the MEWS is a strong predictor of impairment. Furthermore, as discussed above MW may also be measured more objectively using experience sampling methods in daily life or during experimental paradigms. Measures of MW may therefore assist in the accurate diagnosis of individuals based on their mental state rather than descriptions of behavior, which may be more subject to bias or influenced by an individual’s ability to develop compensatory behavioral strategies.

However, currently we do not know the role that excessive MW, as measured by the MEWS, plays in other clinical disorders. For example, in depression depressive rumination represents another form of MW. Thus, the specificity of the MEWS across common mental health disorders needs to be explored. Therefore, we do not currently recommend the routine use of the MEWS to identify patients with ADHD until the scale has been comprehensively evaluated in other psychiatric disorders with overlapping clinical features, although high MEWS scores could be used to support the diagnosis. Investigation of the role of excessive MW in childhood and early adolescent ADHD is also recommended, including use of the scale in this population. Whether children and young adolescents would be able to conceptualize MW and reliably report on their mental state requires investigation.

### Limitations and Future Research

Some participants in Study 2 presented with co-occurring anxiety and depression, raising the possibility that MW might be linked to comorbid conditions. However, in Study 1 participants were free from co-occurring disorders (Skirrow & Asherson, 2013), yet similar results were found. Nevertheless, TUTs are a common feature of most mental health disorders and future research will need to investigate the distinction of excessive MW in ADHD from depressive ruminations, anxious worrying, and other sources of MW.

In relation to ADHD, a key question is whether MW differs conceptually from the inattentive symptoms currently used to define the disorder or whether the mental phenomenon of MW underlies the behavioral expression of inattention. As discussed above, it is feasible that measures of MW in ADHD are a more direct reflection of the neurobiology, leading to the inattentive symptoms of ADHD. Further work is required to evaluate the plausible hypothesis that aberrant regulation of DMN activity linked to excessive MW leads to ADHD symptoms and impairments. The study of MW has several potential advantages over behavioral inattention for research, because it may be measured using rating scales, as reported here, as well as experience sampling during daily life (Killingsworth & Gilbert, 2010), or during sustained attention tasks (Shaw & Giambra, 1993) and neuroimaging studies (Baird, Smallwood, Lutz, & Schooler, 2014; Christoff, Gordon, Smallwood, Smith, & Schooler, 2009).

A fruitful next step in this research will be to take the MEWS into experimental paradigms. For example, an experimental trial of methylphenidate could be used to formally evaluate whether improvements in MW mediate improvements in ADHD symptoms and impairments, and to investigate the underlying neural mechanisms. Yet, currently, there are very little data that link rating scale measures of MW to experimentally derived measures in ADHD. Validation of MW in ADHD is therefore required across the various levels of measurement (rating scale, experience sampling, and experimental paradigms including neuroimaging studies). We hypothesize that MW is a phenomenon that can be reliably measured, and it will be highly informative to see to what extent MEWS scores reflect TUTs measured during cognitive task performance in ADHD. It will also be advantageous to see how it relates to various cognitive measures such as omission and commission errors (Losier, McGrath, & Klein, 1996), and RTV (Kofler et al., 2013), which may reveal further information about the underlying neurobiology of ADHD.

## Conclusion

This research provides further insight into the mental phenomenon of MW in ADHD. We investigate a questionnaire-based measure of excessive MW recently developed in our research group. The MEWS was found to be a valid and reliable measure, with comparable sensitivity and specificity for case-control differences as existing rating scale measures of core ADHD symptoms currently used in clinical practice. The MEWS functioned extremely well for a brief 15-item measure and is potentially a useful measure to incorporate in future clinical and etiological research. MEWS scores were found to be a particularly good predictor of impairment, highlighting the clinical utility of the tool for diagnosis and treatment. Based on these findings, there is strong premise to view MW as a common co-occurring feature of adult ADHD with a specific effect on impairment, potentially explaining a variety of deficits not easily accounted for by the core symptom dimensions.

## Supplemental Material

Mindwandering_and_ADHD_SupplementaryMaterial_JAD – Supplemental material for Validation of the Mind Excessively Wandering Scale and the Relationship of Mind Wandering to Impairment in Adult ADHD Click here for additional data file. Supplemental material, Mindwandering_and_ADHD_SupplementaryMaterial_JAD for Validation of the Mind Excessively Wandering Scale and the Relationship of Mind Wandering to Impairment in Adult ADHD by Florence D. Mowlem, Caroline Skirrow, Peter Reid, Stefanos Maltezos, Simrit K. Nijjar, Andrew Merwood, Edward Barker, Ruth Cooper, Jonna Kuntsi and Philip Asherson in Journal of Attention Disorders
