# Supplementary material for: Validation of the Mind Excessively Wandering Scale and the Relationship of Mind Wandering to Impairment in Adult ADHD
Source: J Atten Disord. 2016 Jun 2;23(6):624–34. doi: 10.1177/1087054716651927 (PMC6429624; doi:10.1177/1087054716651927)
Supplement: Mindwandering_and_ADHD_SupplementaryMaterial_JAD – Supplemental material for Validation of the Mind Excessively Wandering Scale and the Relationship of Mind Wandering to Impairment in Adult ADHD [file Mindwandering_and_ADHD_SupplementaryMaterial_JAD.docx]

**SUPPLEMENTARY MATERIAL**

**Recruitment process for the OCEAN study (Study 2)**

***Controls***

Controls were recruited via recruitment circulars and advertisements in the local community.

***Cases***

Recruitment occurred through four sources:

**1. Recruitment through South London and Maudsley NHS Trust (SLaM):** The medical records of patients (either follow-up patients or those on the waiting list) from the SLaM Adult ADHD Service were screened for eligibility, using the inclusion and exclusion criteria, by a member of the OCEAN research team who held honorary clinical contracts. In addition previous ADHD study databases were also screened for suitable participants. Those deemed eligible were sent study information sheets, invitations, a response slip and a stamped addressed envelope. Where no response slip was returned, participants were contacted by telephone to determine their interest in participating. Those who expressed an interest in participating in the study completed a telephone screening (detailed below). If deemed suitable following the telephone screening and if a Conners Adult ADHD Diagnostic Interview for DSM-IV (CAADID: a structured clinical interview for the 18 ADHD symptoms in childhood and adulthood) (Epstein *et al.* 2001) had been completed as part of their diagnostic assessment at SLaM, then they were invited into the trial and their baseline assessment was booked. If a CAADID were not completed as part of their diagnostic assessment it was completed over the phone by a member of the research team. If the patient was on the waiting list then a research diagnostic assessment was carried out by P. Asherson and R. Copper(detailed below).

**2. Online questionnaire:** In order to recruit undiagnosed patients an online screening questionnaire was set-up (http://neuroknowhow.com/adhdoraddquestionnairepage/) (although this link has now been disabled). This was established by a study participant who runs the website ‘neuroknowhow’ (http://neuroknowhow.com/aboutus/) which provides services and online help for those with neurodevelopmental difficulties such as ADHD, dyslexia, and dyspraxia. The screening questionnaire consisted of the six questions in Part A of the(ASRS) which have been found to be the most predictive of ADHD (Kessler *et al.* 2005). Those who screened above the threshold for ADHD were asked to complete the Barkley Childhood Behaviour Scale. If they scored positive for 6 or more symptoms of either or both domains of inattention or hyperactivity/impulsivity then a research assessment was conducted (see below).

**3. Online advertisements:** Participants were also recruited from advertisements on the ADHD support websites AADD-UK (Adult ADHD-UK) (http://aadduk.org/about/) and ADDISS (The National Attention Deficit Disorder Information and Support Service) (http://www.addiss.co.uk/). We were also contacted from participants who saw the trial registered on clinical trials.gov (identifier: NCT01750307). If these participants had an existing diagnosis we asked them to send us a copy of their diagnostic assessment report. If inclusion/exclusion criteria were met then the CAADID (Epstein *et al.* 2001) was completed by a member of the research team. If the participants did not have an existing diagnosis and screened above threshold for ADHD on the ASRS (Kessler *et al.* 2005) and Barkley’s Childhood Behaviour Scales (Barkley 1998) then a research assessment was conducted (see below).

**4. Recruitment through other doctors:** We attended the clinical team meetings at the Maudsley Adult ADHD Clinic to communicate the study to members of the healthcare team and ask if they had any suitable patients and if they could let their patients know about the study. The study was also circulated to clinicians on the email list of the UK Adult ADHD Network (UKAAN).


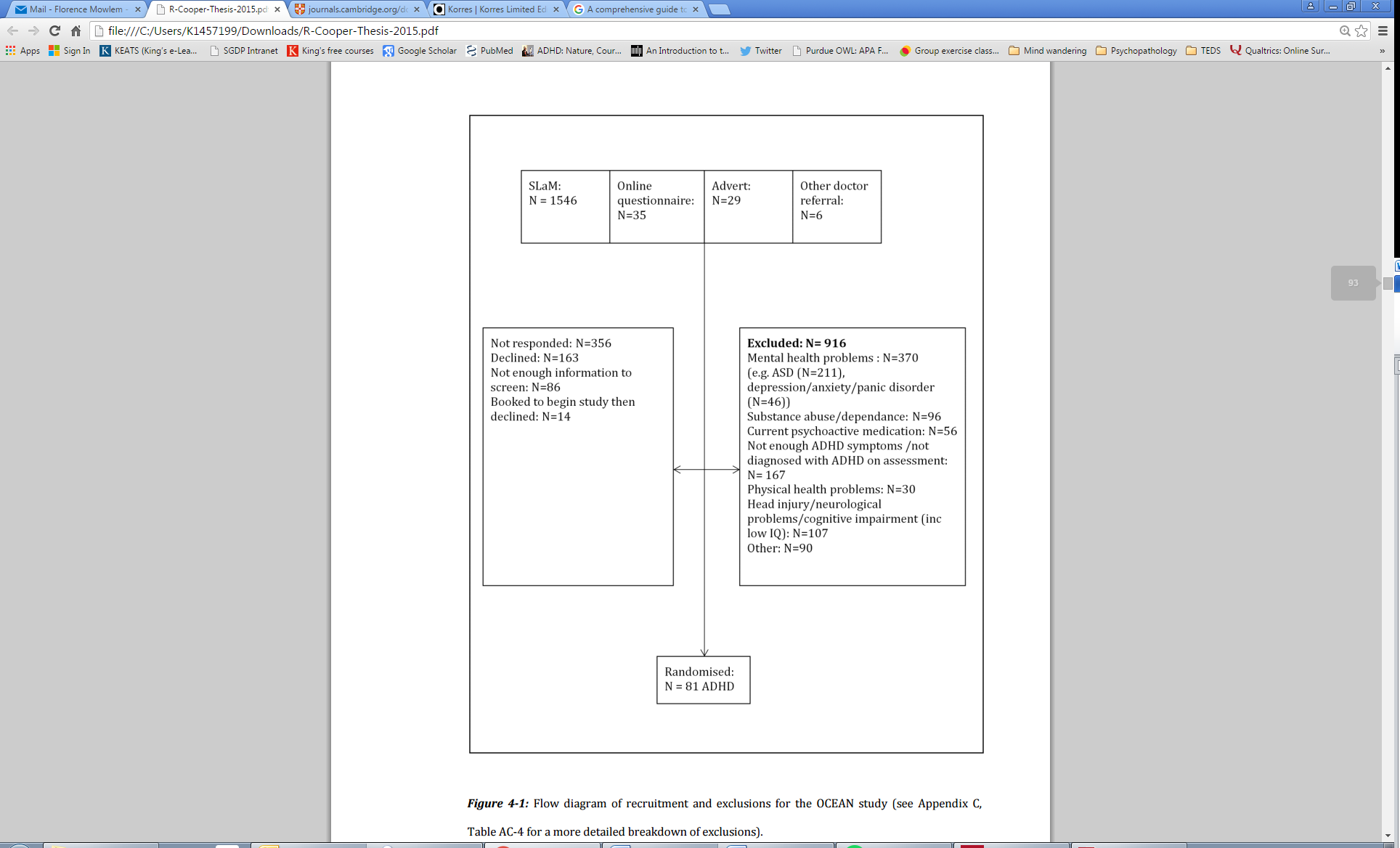


***Telephone screening***

Both ADHD and control participants underwent a structured telephone screening of exclusionary criteria, which consisted of detailed questions assessing any previous or current mental health problems including: presence, treatment for or diagnosis of anxious, depressive and manic/hypomanic symptoms, physical health problems, neurological problems, drinking and drug habits, use of omega-3 or 6 supplements, and any known allergies to fish.

***Research assessment***

Undiagnosed participants who met inclusion/exclusion criteria were asked to complete (over the telephone) the CAADID (Epstein *et al.* 2001). In line with DSM-5, symptom onset and chronicity was established before age 12 and in adulthood, the presence of a minimum of 5 symptoms of inattention and 3 symptoms of hyperactivity/impulsivity were established (*American Psychiatric Associations 2013*). The CAADID was also completed (over the telephone) with someone who knew the participant in childhood, most commonly a parent. The CAADID was then reviewed by P. Asherson, an experienced consultant psychiatrist specialising in adult ADHD, who approved the participants prior to inviting them into the study. In addition, P. Asherson met participants at their baseline assessment to review and confirm the diagnosis. Participants were then provided with a letter from P. Asherson detailing the outcome of the research assessment. Participants who had not yet been referred or diagnosed for adult ADHD could then, if they wished, use this letter to help gain a referral for a formal adult ADHD assessment, although they were asked to not begin medication for the duration of the trial if they wished to take part.

**References**

***American Psychiatric Associations. (2013). Diagnostic and Statistical Manual of Mental Disorders (DSM-5)*** (n.d.). 5th edn. Washington DC: American Psychiatric Association.

**Barkley RA** (1998). *A Clinical Workbook: Attention-Deficit Hyperactivity Disorder*. Guilford, New York.

**Epstein JN, Johnson DE, & Conners CK** (2001). *Conners’ Adult ADHD Diagnostic Interview for DSM-IV.* North Tonawanda, NY: Multi-Health Systems.

**Kessler RC, Adler L, Ames M, Demler O, Faraone S, Hiripi E, Howes MJ, Jin R, Secnik K, Spencer T, Ustun TB, & Walters EE** (2005). The World Health Organization Adult ADHD Self-Report Scale (ASRS): a short screening scale for use in the general population. *Psychological medicine* **35**, 245–256.

**Supplementary Figures:**


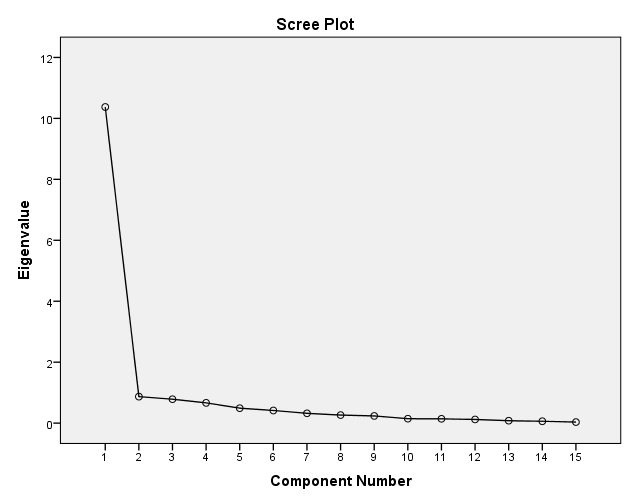


**Supplementary Figure 1.** *Scree plot produced during Factorial Analysis for Study 1 indicating a one-factor solution.*


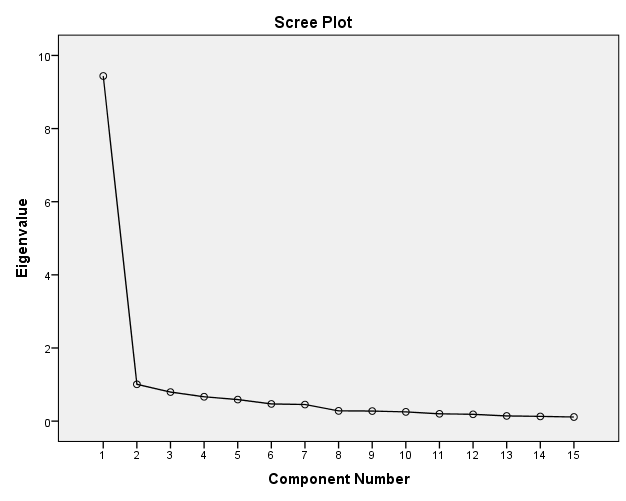


**Supplementary Figure 2.** *Scree plot produced during Factorial Analysis for Study 2 showing a one-factor solution.*

**Supplementary Tables**

**Supplementary Table 1.** *Case-control differences for mind wandering (MEWS), inattention (INN), hyperactivity/impulsivity (HI), emotional lability (EL), and impairment (IMP) at Time 2 and 3*

|  | | ***Study 1*** | | | | | | ***Study 2*** | | | | | | |
| --- | --- | --- | --- | --- | --- | --- | --- | --- | --- | --- | --- | --- | --- | --- |
|  | ADHD | | | Control | | |  | ADHD | | | Control | | |  |
|  | N | M | SD | N | M | SD | *p* | N | M | SD | N | M | SD | *p* |
| ***Time 2*** |  |  |  |  |  |  |  |  |  |  |  |  |  |  |
| MEWS | 24 | 18.87 | 10.15 | 36 | 5.44 | 7.13 | <.0001 | 68 | 24.09 | 10.60 | - | - | - | - |
| INN | 30 | 15.17 | 6.54 | 38 | 3.26 | 3.41 | <.0001 | 68 | 23.25 | 7.91 | - | - | - | - |
| HI | 29 | 11.76 | 6.00 | 38 | 2.71 | 2.78 | <.0001 | 68 | 17.10 | 6.36 | - | - | - | - |
| EL | 30 | 39.03 | 10.84 | 39 | 23.26 | 9.46 | <.0001 | 68 | 21.56 | 10.65 | - | - | - | - |
| IMP | 30 | 0.87 | 0.41 | 38 | 0.26 | 0.29 | <.0001 | 68 | 0.95 | 0.50 | - | - | - | - |
| ***Time 3*** |  |  |  |  |  |  |  |  |  |  |  |  |  |  |
| MEWS | - | - | - | - | - | - | - | 55 | 24.62 | 11.10 | - | - | - | - |
| INN | - | - | - | - | - | - | - | 54 | 23.65 | 7.52 | - | - | - | - |
| HI | - | - | - | - | - | - | - | 54 | 17.11 | 6.43 | - | - | - | - |
| EL | - | - | - | - | - | - | - | 55 | 20.58 | 11.69 | - | - | - | - |
| IMP | - | - | - | - | - | - | - | 55 | 0.90 | 0.51 | - | - | - | - |

**Supplementary Table 2.** *Case-control differences for each domain of impairment at each time point*

|  | | | ***Study 1*** | | | | | | ***Study 2*** | | | | | | |
| --- | --- | --- | --- | --- | --- | --- | --- | --- | --- | --- | --- | --- | --- | --- | --- |
|  | ADHD | | | | Control | | |  | ADHD | | | Control | | |  |
| ***Time 1*** | N | M | | SD | N | M | SD | *p* | N | M | SD | N | M | SD | *p* |
| Family Life | 41 | 1.04 | | 0.66 | 47 | 0.25 | 0.34 | <.0001 | 80 | 1.17 | 0.76 | 30 | 0.23 | 0.30 | <.0001 |
| Work | 39 | 1.31 | | 0.63 | 39 | 0.22 | 0.39 | <.0001 | 68 | 1.27 | 0.74 | 26 | 0.11 | 0.19 | <.0001 |
| School | 31 | 1.90 | | 0.61 | 38 | 0.37 | 0.46 | <.0001 | 40 | 1.88 | 0.64 | 10 | 0.18 | 0.28 | <.0001 |
| Life-Skills | 47 | 1.47 | | 0.53 | 41 | 0.40 | 0.40 | <.0001 | 80 | 1.50 | 0.64 | 30 | 0.27 | 0.25 | <.0001 |
| Self-Concept | 47 | 1.69 | | 0.87 | 41 | 0.56 | 0.55 | <.0001 | 80 | 1.78 | 0.92 | 30 | 0.43 | 0.54 | <.0001 |
| Social | 47 | 1.01 | | 0.59 | 41 | 0.20 | 0.28 | <.0001 | 80 | 1.09 | 0.69 | 30 | 0.12 | 0.19 | <.0001 |
| Risk Taking | 47 | 0.65 | | 0.49 | 41 | 0.23 | 0.26 | =.001 | 80 | 0.64 | 0.46 | 30 | 0.16 | 0.19 | <.0001 |
| ***Time 2*** |  |  | |  |  |  |  |  |  |  |  |  |  |  |  |
| Family Life | 30 | 0.59 | | 0.44 | 38 | 0.24 | 0.39 | <.0001 | 67 | 0.85 | 0.70 | - | - | - | - |
| Work | 27 | 1.04 | | 0.56 | 24 | 0.17 | 0.26 | <.0001 | 54 | 0.91 | 0.68 | - | - | - | - |
| School | 20 | 1.56 | | 0.74 | 24 | 0.32 | 0.51 | <.0001 | 26 | 1.41 | 0.79 | - | - | - | - |
| Life-Skills | 30 | 1.00 | | 0.55 | 38 | 0.40 | 0.51 | <.0001 | 68 | 1.27 | 0.62 | - | - | - | - |
| Self-Concept | 29 | 1.30 | | 0.79 | 38 | 0.51 | 0.52 | <.0001 | 68 | 1.49 | 0.98 | - | - | - | - |
| Social | 29 | 0.71 | | 0.48 | 38 | 0.18 | 0.25 | <.0001 | 68 | 0.82 | 0.62 | - | - | - | - |
| Risk Taking | 29 | 0.40 | | 0.38 | 38 | 0.18 | 0.21 | =.001 | 68 | 0.49 | 0.43 | - | - | - | - |
| ***Time 3*** |  |  | |  |  |  |  |  |  |  |  |  |  |  |  |
| Family Life | - | - | | - | - | - | - | - | 54 | 0.81 | 0.70 | - | - | - | - |
| Work | - | - | | - | - | - | - | - | 42 | 0.89 | 0.73 | - | - | - | - |
| School | - | - | | - | - | - | - | - | 16 | 1.33 | 0.88 | - | - | - | - |
| Life-Skills | - | - | | - | - | - | - | - | 55 | 1.23 | 0.65 | - | - | - | - |
| Self-Concept | - | - | | - | - | - | - | - | 55 | 1.33 | 0.92 | - | - | - | - |
| Social | - | - | | - | - | - | - | - | 55 | 0.80 | 0.68 | - | - | - | - |
| Risk Taking | - | - | | - | - | - | - | - | 55 | 0.46 | 0.37 | - | - | - | - |

**Supplementary Table 3.** *ROC Analysis curve coordinates showing sensitivity and specificity of the 15-item MEWS in the MIRIAD study. Optimum balance of sensitivity (.88) and specificity (.88) is at threshold 15.00*

| **Test result** | **Sensitivity** | **1 - Specificity** |
| --- | --- | --- |
| -1.00 | 1.000 | 1.000 |
| .50 | 1.000 | .667 |
| 1.50 | 1.000 | .583 |
| 2.50 | .920 | .542 |
| 3.50 | .880 | .292 |
| 5.00 | .880 | .250 |
| 6.50 | .880 | .208 |
| 10.50 | .880 | .167 |
| 15.00 | .880 | .125 |
| 16.50 | .840 | .125 |
| 18.50 | .800 | .083 |
| 22.00 | .800 | .042 |
| 24.50 | .680 | .042 |
| 25.50 | .600 | .000 |
| 27.00 | .440 | .000 |
| 28.50 | .400 | .000 |
| 29.50 | .360 | .000 |
| 30.50 | .280 | .000 |
| 32.00 | .240 | .000 |
| 33.50 | .160 | .000 |
| 35.00 | .120 | .000 |
| 37.50 | .040 | .000 |
| 40.00 | .000 | .000 |

**Supplementary Table 4.** *ROC Analysis curve coordinates showing sensitivity and specificity of the 15-item MEWS in the OCEAN study. Optimum balance of sensitivity (.90) and specificity (.90) is at threshold 15.00*

| **Test result** | **Sensitivity** | **1 - Specificity** |
| --- | --- | --- |
| -1.00 | 1.000 | 1.000 |
| .50 | 1.000 | .931 |
| 1.50 | 1.000 | .759 |
| 2.50 | 1.000 | .724 |
| 3.50 | 1.000 | .655 |
| 4.50 | 1.000 | .621 |
| 5.50 | .987 | .552 |
| 7.00 | .987 | .379 |
| 8.50 | .987 | .345 |
| 9.50 | .987 | .310 |
| 10.50 | .975 | .241 |
| 11.50 | .949 | .207 |
| 13.00 | .924 | .207 |
| 14.50 | .911 | .138 |
| 15.50 | .899 | .103 |
| 17.00 | .848 | .103 |
| 18.50 | .810 | .069 |
| 19.50 | .785 | .069 |
| 20.50 | .759 | .069 |
| 21.50 | .759 | .034 |
| 22.50 | .734 | .000 |
| 23.50 | .684 | .000 |
| 24.50 | .671 | .000 |
| 25.50 | .633 | .000 |
| 26.50 | .582 | .000 |
| 27.50 | .557 | .000 |
| 28.50 | .443 | .000 |
| 29.50 | .418 | .000 |
| 30.50 | .354 | .000 |
| 31.50 | .329 | .000 |
| 32.50 | .304 | .000 |
| 33.50 | .278 | .000 |
| 34.50 | .253 | .000 |
| 35.50 | .215 | .000 |
| 37.00 | .177 | .000 |
| 38.50 | .152 | .000 |
| 39.50 | .139 | .000 |
| 40.50 | .127 | .000 |
| 41.50 | .076 | .000 |
| 42.50 | .063 | .000 |
| 43.50 | .025 | .000 |
| 45.00 | .000 | .000 |

**Supplementary Table 5.** *ROC Analysis curve coordinates showing sensitivity and specificity of the 12-item MEWS in the OCEAN study. Optimum balance of sensitivity (.89) and specificity (.90) is at threshold 15.00*

| **Test result** | **Sensitivity** | **1 - Specificity** |
| --- | --- | --- |
| -1.00 | 1.000 | 1.000 |
| .50 | 1.000 | .897 |
| 1.50 | 1.000 | .759 |
| 2.50 | 1.000 | .724 |
| 4.00 | 1.000 | .483 |
| 6.50 | .987 | .345 |
| 8.50 | .975 | .276 |
| 9.50 | .937 | .207 |
| 10.50 | .924 | .172 |
| 11.50 | .911 | .138 |
| 12.50 | .886 | .103 |
| 13.50 | .873 | .103 |
| 14.50 | .848 | .103 |
| 15.50 | .835 | .069 |
| 16.50 | .797 | .069 |
| 17.50 | .785 | .000 |
| 18.50 | .759 | .000 |
| 19.50 | .722 | .000 |
| 20.50 | .684 | .000 |
| 21.50 | .646 | .000 |
| 23.00 | .570 | .000 |
| 24.50 | .494 | .000 |
| 25.50 | .430 | .000 |
| 27.00 | .342 | .000 |
| 28.50 | .316 | .000 |
| 29.50 | .266 | .000 |
| 30.50 | .215 | .000 |
| 31.50 | .177 | .000 |
| 32.50 | .152 | .000 |
| 33.50 | .127 | .000 |
| 34.50 | .114 | .000 |
| 35.50 | .063 | .000 |
| 37.00 | .000 | .000 |

**Supplementary Table 6.** *Cross-scale correlations between the mind wandering (MEWS), inattention (INN), hyperactivity/impulsivity (HI), emotional lability (EL), and impairment (IMP) rating scales in cases and controls*

| Study 1 | | | | | | | | |
| --- | --- | --- | --- | --- | --- | --- | --- | --- |
| Time 1 | INN | | HI | | EL | | IMP | |
|  | Cases | Controls | Cases | Controls | Cases | Controls | Cases | Controls |
| MEWS | 0.50* | 0.87*** | 0.38 | 0.81*** | 0.63** | 0.74*** | 0.56** | 0.75*** |
| INN |  |  | 0.62*** | 0.77*** | 0.48** | 0.66*** | 0.58*** | 0.68*** |
| HI |  |  |  |  | 0.61*** | 0.64*** | 0.59*** | 0.69*** |
| EL |  |  |  |  |  |  | 0.53*** | 0.77*** |
| Study 2 | | | | | | | | |
| Time 1 | INN | | HI | | EL | | IMP | |
|  | Cases | Controls | Cases | Controls | Cases | Controls | Cases | Controls |
| MEWS | 0.50*** | 0.38* | 0.36** | 0.23 | 0.58*** | 0.65*** | 0.67*** | 0.56** |
| INN |  |  | 0.46*** | 0.35 | 0.23** | 0.52** | 0.46*** | 0.57** |
| HI |  |  |  |  | 0.13 | 0.35 | 0.23* | 0.59** |
| EL |  |  |  |  |  |  | 0.62*** | 0.56** |

* *p* < .05, ** *p* < .01, *** *p* <.001
